# Evaluation of right ventricular performance in patients with postoperative congenital heart disease using Doppler tissue imaging and cardiopulmonary bypass indices: A prospective cohort study

**DOI:** 10.1002/hsr2.909

**Published:** 2022-10-29

**Authors:** Vishal V. Bhende, Tanishq S. Sharma, Bhadra Y. Trivedi, Amit Kumar, Dushyant M. Parmar, Paresh Nerurkar, Prachi M. Shah, Naresh J. Fumakiya, Hardil P. Majmudar, Sohilkhan R. Pathan

**Affiliations:** ^1^ Department of Pediatric Cardiac Surgery, Bhanubhai and Madhuben Patel Cardiac Centre, Shree Krishna Hospital Bhaikaka University Gujarat India; ^2^ Department of Pediatric Cardiology, Bhanubhai and Madhuben Patel Cardiac Centre, Shree Krishna Hospital Bhaikaka University Gujarat India; ^3^ Department of Pediatric Cardiac Intensive Care, Bhanubhai and Madhuben Patel Cardiac Centre, Shree Krishna Hospital Bhaikaka University Gujarat India; ^4^ Department of Perfusion Technology, Bhanubhai and Madhuben Patel Cardiac Centre, Shree Krishna Hospital Bhaikaka University Gujarat India; ^5^ Department of Echocardiography, Bhanubhai and Madhuben Patel Cardiac Centre, Shree Krishna Hospital Bhaikaka University Gujarat India; ^6^ Clinical Research Coordinator, Central Research Services (Crs), Bhanubhai and Madhuben Patel Cardiac Centre, Shree Krishna Hospital Bhaikaka University Gujarat India

**Keywords:** cardiopulmonary bypass, congenital heart disease, Doppler tissue imaging, intracardiac repair, ventricular performance

## Abstract

**Background and Aims:**

Postoperative cardiac outcomes after intracardiac repair (ICR) are determined by numerous factors whereas right ventricle (RV) dysfunction is considered essential for them, as only few studies attempted to evaluate it postsurgically. RV's function is supposed to be the strong prognostic factor for patients diagnosed with congenital heart defects; therefore, assessing it is the main objective of the study.

**Methods:**

This is a prospective single‐centered cohort study performed on 50 pediatric patients with congenital heart disease (CHD) who underwent ICR between January 2019 and January 2022. All patients underwent echocardiographic assessment of RV function via tricuspid annular plane systolic excursion (TAPSE) and fractional area change (FAC) at 1, 24, and 48 h. After surgery, where pre‐ and postoperative RV pressure, cardiopulmonary bypass (CPB), and aortic cross‐clamp (ACC) time were assessed. Similarly ventilation intensive care unit (ICU) and hospital stay times and mediastinal drainage were also monitored.

**Results:**

The mean ± standard deviation for pre‐ and postoperative RV pressure was 49.1 ± 16.12 and 42.7 ± 2.9 mmHg, respectively, whereas that for pre‐ and postoperative pulmonary artery pressure was 30.4 ± 2.6 and 24.2 ± 12.9 mmHg, with *p* value of <0.002 and <0.001, respectively. The mean ± standard deviation of CPB and ACC times was 120.92 ± 74.17 and 78.44 ± 50.5 min accordingly, while those for mean ± standard deviation of ventilation time, mediastinum chest drainage, ICU and hospital stays were 30.36 ± 54.04, 43.78 ± 46.7 min, 5.9 ± 4.01 h, were 30.36 ± 54.0, 43.78 ± 46.7 min, 5.9 ± 4.01 and 10.3 ± 4.83 h, respectively.

**Conclusions:**

RV dysfunction plays the important role in longer recovery and intraoperative time, while its effect is mostly transient. The use of TAPSE and FAC methods is valuable in the evaluation of postoperative outcomes, and the former proved to be more effective.

## 1 INTRODUCTION

Cardiopulmonary bypass (CPB) is a surgical procedure that is commonly used in clinical practice, particularly for the treatment of congenital heart diseases (CHDs) in the pediatric group of patients. One of the most serious complications associated with it is right ventricle (RV) dysfunction that can lead to profound ischemia and myocardial infarction.^1^

CPB guarantees a motionless and bloodless surgical field by involving an extracorporeal circuit to provide physiological support. Typically, blood is drained by gravity out of the heart and lungs to a reservoir by venous cannulation and tubing. Later, a pump and artificial lung (oxygenator or gas exchanger) facilitate the return of the oxygenated blood to the arterial system.

CPB is a surgical intervention that significantly affects systemic metabolism and endocrine function. Many trials have attempted and tried to assess the predictive value of fatty acid‐binding protein compared with other biomedical markers to predict perioperative myocardial injury and early ventricular dysfunction.

Conversely, on the other hand, most interventions with CPB with cardioplegic arrest lead to ischemic–reperfusion injury resulting in myocardial dysfunction. This dysfunction may be temporary (up to 24 h), stunning, and even persistent in profound ischemia and myocardial infarction cases. However, there are many contributing factors such as preoperative myocardial dysfunction, degree of myocardial protection, systemic inflammatory responses, and alterations in signaling transduction systems.

RV dysfunction pathology includes high RV preload or afterload, impaired right coronary artery perfusion, and decreased contractility. In contrast to the left coronary artery, perfusion of the right coronary artery occurs during the whole heart cycle. Moreover, high RV pressure due to pathological causes such as pulmonary hypertension leads to lower coronary artery perfusion, highlighting the importance of preserving the normal pressure inside the two ventricles. Additionally, the RV normally provides low‐pressure perfusion for the pulmonary vasculature, making it highly sensitive to even moderate increases in pulmonary arterial pressure. However, RV dysfunction is usually a result of different mechanisms, and that is why RV failure is associated with many underlining conditions. Contractility impairment as one of the major causes of RV dysfunction is mainly caused by perioperative RV ischemia and infarction. Volume overload, on the other hand, is commonly developed as a result of tricuspid or pulmonic regurgitation leads. However, the causes of pressure overload include left‐sided valvular disease, cardiomyopathy, pulmonary hypertension, embolism, acute respiratory distress syndrome (ARDS), and high positive‐pressure ventilation. Pulmonary hypertension or contractile impairment may lead to RV failure associated with rapid progression to RV dilation, resulting in higher end‐diastolic pressure. That can push the ventricular septum toward the already underfilled left ventricle (LV) chamber, which, in its turn, reduces LV preload and decreases CO.

The physiopathological process of RV failure after cardiac surgery is more complex than what we have described above in the traditional model. More than one mechanism is involved in the process, including pulmonary hypertension primarily associated with CPB, pre‐existing pulmonary hypertension, and ventricular interdependency. Inflammatory mediators after CPB may mediate the increase in pulmonary resistance and vasoconstriction after CPB. These mediators may result from endothelial damage or ischemic and reperfusion mechanisms due to inadequate blood flow through the bronchial arteries. As a result, nitric oxide and prostacyclin go down, while thromboxane A2 and endothelin level up. This represents the imbalance between vasoconstriction and vasodilation factors. Additional factors disturb the hemodynamic status of RV and increase pulmonary hypertension, such as administration of heparin and/or protamine, pulmonary microembolism phenomena, ischemia of the RV, metabolic acidosis, hypercapnia, hypothermia, hydric overload, poor myocardial protection, extended extracorporeal circulation time, obstruction of vascular grafts, and loss of auricular–ventricular synchrony.

The relevant diagnostic methods are used to prevent these complications, and one of them is echocardiography.

The echocardiographic machine uses ultrasound waves, and it must have some features to perform standard pediatric echocardiograms. These features must include two‐dimensional (2D) imaging, M‐mode, pulsed‐wave and continuous‐wave Doppler, color flow Doppler mapping, electrocardiogram 2D gating, Doppler velocities, and a color monitor. Additionally, it must have the ability to measure cardiac structures and store moving images. A hard‐copy paper printout is desirable but not required.

Although evaluating ventricular function is an essential part of every echocardiographic study on children with congenital or acquired heart disease, it is still challenging to evaluate the systolic function. That is because of the confounding effect of different factors, such as ventricular structure and the difference in loading conditions.

Evaluating contractility, on the other hand, is also difficult because of the complex structure physiology of RV. Many indices have been described as surrogate parameters of RV global systolic function. According to the latest guidelines for cardiac chamber quantification, sonographers recommended observing the right heart from various perspectives using multiple acoustic windows. Additionally, including multiple parameters is strongly needed to give the best result.

During cardiac anesthesia and in the intensive care unit (ICU), clinicians use echocardiography to make the right decisions regarding surgical procedures and perioperative management.^2^ Echocardiography, conversely, helps in assessing the hemodynamic status besides static and dynamic parameters such as low CO syndrome, ejection fraction, heart volumes, systolic and diastolic function, valve pathology, pulmonary circulation, ventricular filling pressures, pericardial effusion, and fluid responsiveness.^2^, ^3^

Thus, considering the prevalence of heart diseases in children, the use of echocardiography to assess RV performance during the heart cycle is crucial to determine the long‐term outcomes of surgery and to take the appropriate intervention in case of poor RV performance, especially if there are congenital diseases present. Therefore, RV evaluation using the appropriate measurements should be performed routinely using various parameters according to the main diagnosis.

In modern clinical practice, the evaluation of RV systolic function is routinely implemented via Doppler tissue imaging, tricuspid annular plane systolic excursion (TAPSE), derived tricuspid lateral annular systolic velocity (S wave), and fractional area change (FAC).

## 2 FAC

**Figure 1:**
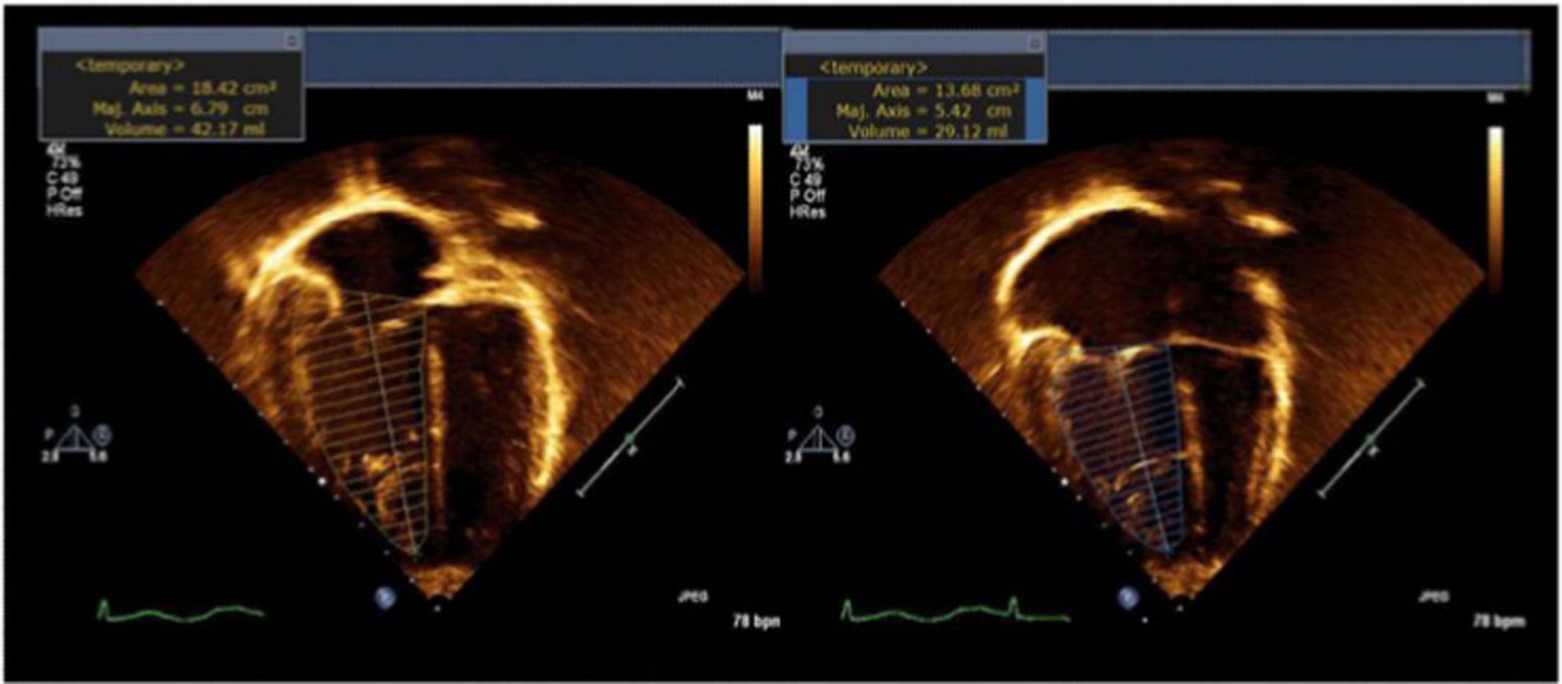
The change of fractional area was calculated by delineating the RV endocardial border at the end‐diastole and end‐systole phases. The difference between the two areas was divided by the area at end‐diastole. However, this measure is a reproducible one that is unaffected by pericardiotomy. The normal change for adults is greater than 35%. In this example, it reduced to (26%), which refers to an RV dysfunction. RV, right ventricle/right ventricular.

The FAC is a 2D measure of RV global systolic function where the normal value is >35% in adults.^4^ Using the apical four‐chamber view, it is calculated by subtracting the end‐diastolic from the end‐systolic area and then dividing the result by the end‐diastolic area.^5^ The image must be optimized under two essential conditions: maximizing the RV area and defining the border of the endocardium in the setting of trabeculations, especially the free wall, to accurately capture the RV cavity.^6^ According to the previous studies, FAC correlates with magnetic resonance imaging (MRI)‐derived RV ejection fraction (RVEF). Additionally, it can predict the outcomes in adult patients with myocardial infarction and pulmonary hypertension.^7^, ^8^ Contrary, in contrast to the other RV measures such as TAPSE, FAC is preserved after pericardiotomy. Therefore, it is preferred over other methods to assess postoperative RV function in case of an altered RV contractile pattern. Although some studies have reported the use of FAC in CHD, this was not true for most cases. In patients with surgically repaired Tetralogy of Fallot, for example, the RV outflow tract is patched and often dysfunctional. However, FAC may overestimate the global RV function in this group of patients because it does not consider the contribution of the RV outflow tract to ejection.^9^ By contrast, FAC demonstrates a good correlation with EF as measured by cardiac MRI (CMRI) in adult patients with repaired tetralogy of Fallot, which was reproducible in many adult studies.^10^ Conversely, EF shows a poor correlation with other measures such as TAPSE, tricuspid S′ velocity, and myocardial performance index. Consequently, in this population, FAC can predict impaired RV function before and after pulmonary valve replacement.^11^ Despite the lack of normative values for the pediatric population, FAC should be implemented in pediatric studies after assuring an adequate four‐chamber view and proper tracing of the endocardial borders^12^ (Table 1, Figure 1).

**Table 1:** RV FAC (%)

Reference range	32%–60%
Mild abnormal	25%–31%
Moderate abnormal	18%–24%
Severe abnormal	<17%

Abbreviations: FAC, fractional area change; RV, right ventricle/right ventricular.

## 3 TAPSE

**Figure 2:**
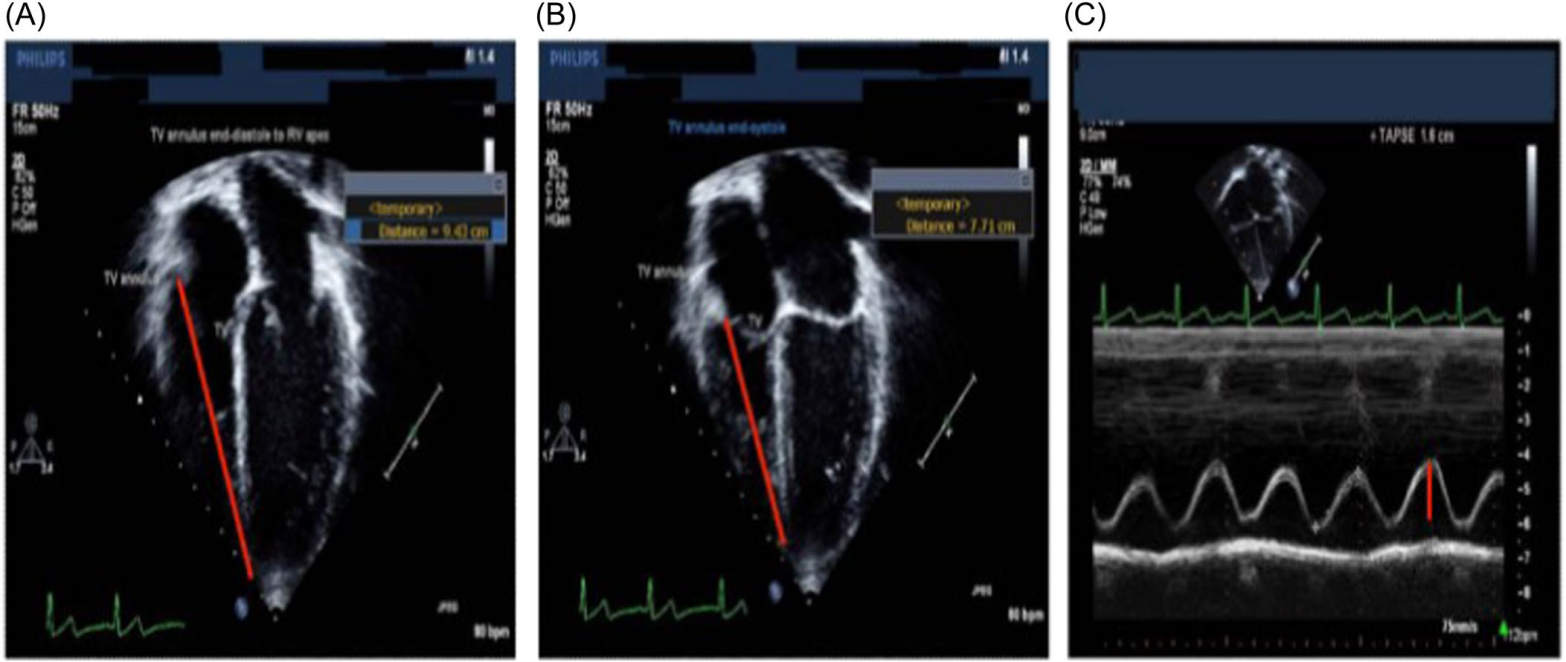
TAPSE is determined by using 2D imaging (A, B) or placing the M‐mode cursor at the level of the tricuspid valve annulus (C). 2D, two‐dimensional; TAPSE, tricuspid annular plane systolic excursion.

TAPSE is another 2D measuring procedure that evaluates systolic RV function in the M‐mode apical four‐chamber view by placing the cursor on the lateral section of the tricuspid valve annulus. It assesses the tricuspid valve's excursion by calculating the difference in distance between the annulus and the apex at the end‐diastole and end‐systole phases. This distance can also be easily quantified with 2D imaging techniques,^13^, ^14^ with equally repeatable findings. Conversely, the load and angle dependence besides the potential influence of the LV's functional status represent big limitations for the procedure. Furthermore, TAPSE does not consider the involvement of the ventricular septum and/or the RV outflow tract in the performance of RV.^15^ Considering the pathological change in the contractile pattern of the RV, this measure poorly correlates with MRI‐measured EF. Therefore, it should be cautiously used in pediatric patients with RV volume load and single ventricle and is preferably reserved for longitudinal follow‐up.^14^, ^16^, ^17^ Similar to adults, TAPSE is especially effective in children with pulmonary arterial hypertension, where values of >15 mm. are related to a three‐fold event rate compared with those with normal values.^13^, ^18^, ^19^ Normal values according to age are available for different life stages such as the premature, neonatal, and pediatric populations (Table 2, Figure 2).

**Table 2:** TAPSE (mm)

Normal	>15 mm
Mild RV dysfunction	14–15 mm
Moderate RV dysfunction	10–14 mm
Severe RV dysfunction	<10 mm

Abbreviations: RV, right ventricle/right ventricular; TAPSE, tricuspid annular planar systolic excursion.

Electrocardiogram (ECG) tracing can be used to determine the optimal frame to measure the longitudinal shortening of the RV. The normal value in adults is greater than 1.6 cm, which is different from that of children. TAPSE, conversely on the other hand, can assess RV function effectively in patients with pulmonary hypertension or those with nonoperated hearts. Despite the reproducibility of the measure, it correlates poorly with EF compared with MRI measures. Therefore, it should be cautiously used in congenital heart defects that involve RV, such as single and systemic RVs, and Tetralogy of Fallot.

As RV function plays a crucial role in the outcomes of patients who undergo cardiac surgery, our study aims to compare the efficacy of TAPSE and FAC in the postoperative measurement of RV function. This also includes an investigation of the influence of the surgical approach and its correlation with CPB.

## 4 MATERIALS AND METHODS

This is a prospective observational study on pediatric patients with CHD who underwent cardiac surgery at Bhanubhai and Madhuben Patel Cardiac Centre, Shree Krishna Hospital, Karamsad, Anand, Gujarat, India, between January 2019 and January 2020.

A total of 50 patients were enrolled, and the following inclusion criteria were applied: age between 1 month and 18 years and the presence of CHD. Exclusion criteria comprised consisted of preoperative moderate‐to‐severe RV dysfunction, single ventricle physiology, an abnormal left coronary artery from the PA (ALCAPA), and LV dysfunction besides moderate‐to‐severe RV dysfunction (Table 3).

**Table 3:** Master chart of the patients participating in the study

Sr. No.	Age (months)	Sex	Height (cm)	Body surface area	Hb (g%)	ACC (min)	CPB (min)	RV pressure (mmHg) (preoperative)	RV pressure (mmHg) (postoperative)	MPA pressure (mmHg) (preoperative)	MPA pressure (mmHg) (postoperative)	Ventilation (h)	Drain time (h)	ICU stay (days)	Hospital stay (days)	TAPSE baseline (preoperative)	FAC after 1 h	FAC after 24 h	FAC after 48 h	FAC baseline (preoperative)	TAPSE after 1 h	TAPSE after 24 h	TAPSE after 48 h
l	60	2	102	0.62	12.3	32	52	65	66	17	14	12	22	4	7	30	25	27	30	13	17	14	14
2	10	2	60	0.35	10.5	131	186	13	40	13	18	23	45	9	13	34	29	29	30	17	10	10	15
3	4	1	53	0.23	10	227	305	42	49	67	30	336	286	18	22	35	30	30	32	16	13	13	14
4	10	1	63	0.33	13.5	35	50	70	52	49	31	14	25	5	8	40	30	30	35	17	12	12	15
5	48	2	95	0.56	14.3	61	83	63	61	49	62	18	30	7	10	35	29	29	30	15	8	8	11
6	4	1	85	0.5	15.3	127	185	43	31	10	14	120	72	9	13	35	25	27	35	14	8	5	7
7	7	1	114	0.75	18.6	139	186	47	30	18	20	22	72	4	9	40	25	33	35	15	6	10	14
8	120	1	124	0.74	12.9	216	300	51	39	16	21	16	63	4	9	30	25	28	30	12	7	8	10
9	5	1	68	0.33	10.4	133	197	46	24	23	18	120	149	17	22	40	27	30	30	16	8	11	6
10	84	2	102	0.6	13.6	67	97	23	20	42	35	18	30	7	11	32	28	28	29	14	13	12	12
11	5	1	61	0.28	15	183	327	40	23	15	17	22	44	8	12	40	25	33	35	17	8	10	14
12	96	1	116	0.76	13.9	61	88	45	32	64	43	12	22	4	7	35	25	29	31	15	8	10	10
13	108	2	122	0.7	11	36	52	68	70	24	22	7	14	2	5	35	31	33	34	13	10	12	12
14	12	1	75	0.41	14.5	59	205	36	34	55	48	18	27	7	11	45	29	29	32	18	8	10	12
15	2	1	52	0.21	15	130	228	40	23	15	17	23	45	22	30	40	26	27	30	16	7	8	11
16	9	1	74	0.37	13.6	162	217	63	32	15	21	24	74	8	12	33	28	28	31	15	11	10	11
17	108	1	116	0.61	13.3	118	166	44	35	14	20	48	81	7	12	30	25	27	32	12	8	10	11
18	24	2	87	0.5	12	37	54	73	69	21	18	8	12	3	4	32	25	25	27	12	8	9	12
19	4	2	56	0.26	10	35	55	58	57	19	19	7	10	3	5	40	27	29	33	14	8	10	11
20	2	1	57	0.26	15	150	216	50	46	22	19	120	144	11	16	42	25	26	31	13	7	10	12
21	3	2	52	0.19	9.7	35	64	49	59	38	19	10	48	5	8	30	24	27	28	10	6	7	8
22	12	2	96	0.49	13.3	108	150	45	24	12	12	18	27	5	11	36	24	26	27	16	6	7	11
23	5	1	60	0.26	9.6	48	70	61	60	59	29	8	46	5	12	35	24	26	31	13	8	7	11
24	84	2	112	0.7	11.8	50	97	74	60	70	51	48	59	5	11	40	20	22	27	15	6	6	8
25	96	1	118	0.78	12.8	77	112	22	13	23	14	20	28	8	13	35	25	29	31	15	10	12	12
26	12	2	67	0.29	14	138	245	63	48	56	44	16	20	5	9	33	28	29	31	14	10	11	13
27	3	1	56	0.23	11.5	67	91	53	55	34	26	9	34	5	7	36	25	29	31	15	8	10	12
28	96	1	118	0.77	13	61	86	62	64	20	26	9	15	2	4	33	27	29	31	13	8	10	12
29	48	1	97	0.5	11	38	65	47	78	15	18	9	20	4	5	33	25	27	28	14	9	10	13
30	6	1	63	0.28	10	45	79	24	22	46	24	9	15	5	11	34	27	29	33	15	10	11	12
31	108	2	124	0.73	13	41	61	73	86	22	15	9	12	3	5	36	30	32	35	14	11	11	13
32	12	1	68	0.3	11.5	34	57	36	34	43	33	10	23	6	9	37	29	30	34	14	12	12	13
33	5	1	62	0.29	11.2	48	76	55	54	22	23	9	42	8	10	40	26	29	33	15	10	11	14
34	9	1	71	0.33	10.9	42	70	17	17	39	19	9	40	4	9	37	29	29	33	16	8	10	13
35	12	1	76.5	0.4	10.6	81	117	35	14	19	14	35	48	7	13	30	24	26	29	12	7	9	10
36	10	1	66	0.36	9.3	135	190	35	14	19	14	32	48	5	13	33	23	26	28	14	6	8	12
37	24	2	80	0.42	9.3	45	66	47	16	22	9	9	18	2	7	37	27	29	34	15	8	10	11
38	12	1	69	0.37	9.1	58	92	53	43	13	10	9	18	2	9	30	23	26	28	14	7	8	10
39	4	1	62	0.28	10.7	36	63	29	33	22	28	10	24	2	8	35	28	29	33	15	10	11	13
40	4	2	58	0.26	10.4	48	73	48	55	38	24	9	17	5	8	38	27	29	33	15	10	12	14
41	4	1	62	0.29	11	44	85	60	16	24	9	9	18	3	14	36	25	28	0.3	15	6	8	11
42	24	1	79	0.43	12	31	56	66	75	15	16	9	24	5	8	38	29	31	0.34	17	10	13	15
43	8	2	63	0.32	9.5	50	74	22	14	18	18	9	26	5	8	36	28	29	0.33	14	7	10	12
44	60	1	94	0.45	10	85	125	53	66	88	66	12	30	3	16	30	22	25	0.28	12	6	7	10
45	4	1	58	0.27	10	92	139	40	32	27	27	9	26	5	12	35	27	29	0.31	14	10	11	12
46	120	1	107	0.66	12	51	76	77	70	23	18	10	14	2	5	40	31	33	0.36	15	11	12	14
47	7	2	61	0.27	14	56	91	48	21	58	48	14	26	6	9	40	29	29	0.31	15	8	7	8
48	108	1	121	0.7	12	39	59	73	77	26	21	8	14	2	4	34	29	30	0.33	14	10	11	13
49	48	1	98	0.52	10.5	23	41	68	63	17	20	9	22	4	8	35	27	29	0.33	16	10	11	12
50	3	2	61	0.28	10	77	127	40	23	24	12	144	120	9	11	36	26	27	0.29	14	8	9	10

Abbreviations: ACC, aortic cross‐clamp; CPB, cardiopulmonary bypass; ICU, intensive care unit; MPA, main pulmonary artery; RV, right ventricle/right ventricular; TAPSE, tricuspid annular planar systolic excursion.

### 4.1 Data collection

The study was approved by the institutional ethics committee, and all the participants provided written informed consent. All the operations, including the aorta‐bicaval cannulation, were performed under traditional CPB through median sternotomy. All the surgical procedures were provided access through the right atrium where we cross‐clamped the aorta, all patients were cooled to 28°C, and cold‐blood cardioplegia (CP) was supplied through the aortic root to stop the heart.

### 4.2 Surgical procedure

Anesthesia was induced and maintained by the weight‐related doses of thiopental, fentanyl, midazolam, and pancuronium.

After shifting patients to the operation theater, standard monitoring viz. ECG, pulse oximetry, and invasive blood pressure monitoring, temperature monitoring with the nasopharyngeal probe was done for all the patients enrolled in the study.

Anesthesia induction was done by injecting ketamine 1 mg/kg, fentanyl 2 mcg/kg, and midazolam 0.02–0.05 mg/kg, and orotracheal intubation was facilitated by injecting vecuronium 0.08–0.1 mg/kg.

Routine airway and ventilator management was done. Anesthesia was maintained with oxygen, sevoflurane, and top‐up doses of injected fentanyl, vecuronium, and midazolam.

### 4.3 Postoperative sedatives

In the postoperative period, analgesia and sedation were achieved via dexmedetomidine infusion at a rate of 0.2–0.7 mcg/kg/h. It is titrated as per the pain score. In patients with relative bradycardia, fentanyl/midazolam infusion was used. The dose of fentanyl infusion is at a rate of 0.5–2 mcg/kg/h and the dose of midazolam infusion is at a rate of 1–5 mcg/kg/h.

In all cases, CPB was performed in the standard manner using a nonpulsatile roller pump, membrane oxygenators, and standard (uncoated) extracorporeal circuits under mild hypothermia (28°C). The circuit was primed with appropriate amounts of Plasmalyte‑A solution, mannitol, and sodium bicarbonate. Blood was added as required.

Through the right atrium, the surgeon performed aorto‐bicaval cannulation, cold‐blood root CP (with del Nido solution), and ventricular septal defect repair with a glutaraldehyde‐treated pericardial patch. The right atrium was used to close the ventricular septal defect. Then, a restricted ventriculotomy was done in the RV outflow tract, beginning 0.5 cm below the annulus and extending for 1–2 cm, through which infundibular resection and pulmonary valvotomy were performed. Commisssurotomy (in the presence of commissural fusion) or release of tethering was used to treat pulmonary valve stenosis, as well as dilating the pulmonary valve with an appropriately sized Hegar dilator. The ventriculotomy was subsequently closed by a primary RV outflow tract patch using autologous glutaraldehyde‑fixed pericardium along with a bovine pericardial patch. From either route, the pulmonary annulus was assessed by passing a Hegar dilator expected for that weight if the pulmonary annulus was stenotic and of small size, followed by an incision of the RV outflow tract that extended across the annulus with resection of parietal and septal bands in the RV outflow tract.

### 4.4 Intraoperative and postoperative data

Perioperative data were collected for CPB and aortic cross‑clamp (ACC) times, mean arterial pressure, and RV pressure. After the repair was completed, the pressure in the right ventricle/pressure in the left ventricle was measured in all patients. If there was no gradient between the RV and the PA and the hemodynamics was as steady, the value of 0.8 was accepted. All patients had intraoperative transesophageal echocardiography to assess the repair's adequacy and biventricular function. Hemodynamic status, particularly central venous pressure, cardiac rhythm stability, time to peripheral warming, and attainment of negative fluid balance were all measured postoperatively. We assessed the need for inotropes by calculating the inotropic score using the following formula:

Inotropicscore = dopaminedose(μg/kg/min) +dobutaminedose(μg/kg/min)+100×epinephrinedose(μg/kg/min)+10 × milrinone dose(μg/kg/min)+10,000×vasopressin.

### 4.5 Echocardiographic assessment

A single echocardiographer assessed the patients (using an ACUSON X300™ ultrasound machine with a frequency range of 1.2–13 MHz). As a reference for RV performance, TAPSE was estimated in the apical four‐chamber view using M‐mode echocardiography. The longitudinal excursion of the TAPSE's lateral annulus was found to have a high association with RVEF calculated from isotopic data. FAC was defined as (end‐diastolic area − end‐systolic area)/end‐diastolic area × 10. The values were obtained postoperatively by tracing the RV endocardium both in end‐diastole and end‐systole from the annulus, along the free wall to the apex, and then back to the annulus, along the interventricular septum. We recorded the values preoperatively and in the postoperative period after 1, 24, and 48 h.

### 4.6 Statistical analysis

All the patients' data were entered into Microsoft Excel, and the analysis while it was carried out by STATA 14.2. Descriptive statistics (mean (SD), frequency [%]), where the characteristics of the study participants were described and analyzed with the paired *t*‐test. A *p* value of <0.05 was considered statistically significant.

## 5 RESULTS

**Figure 3:**
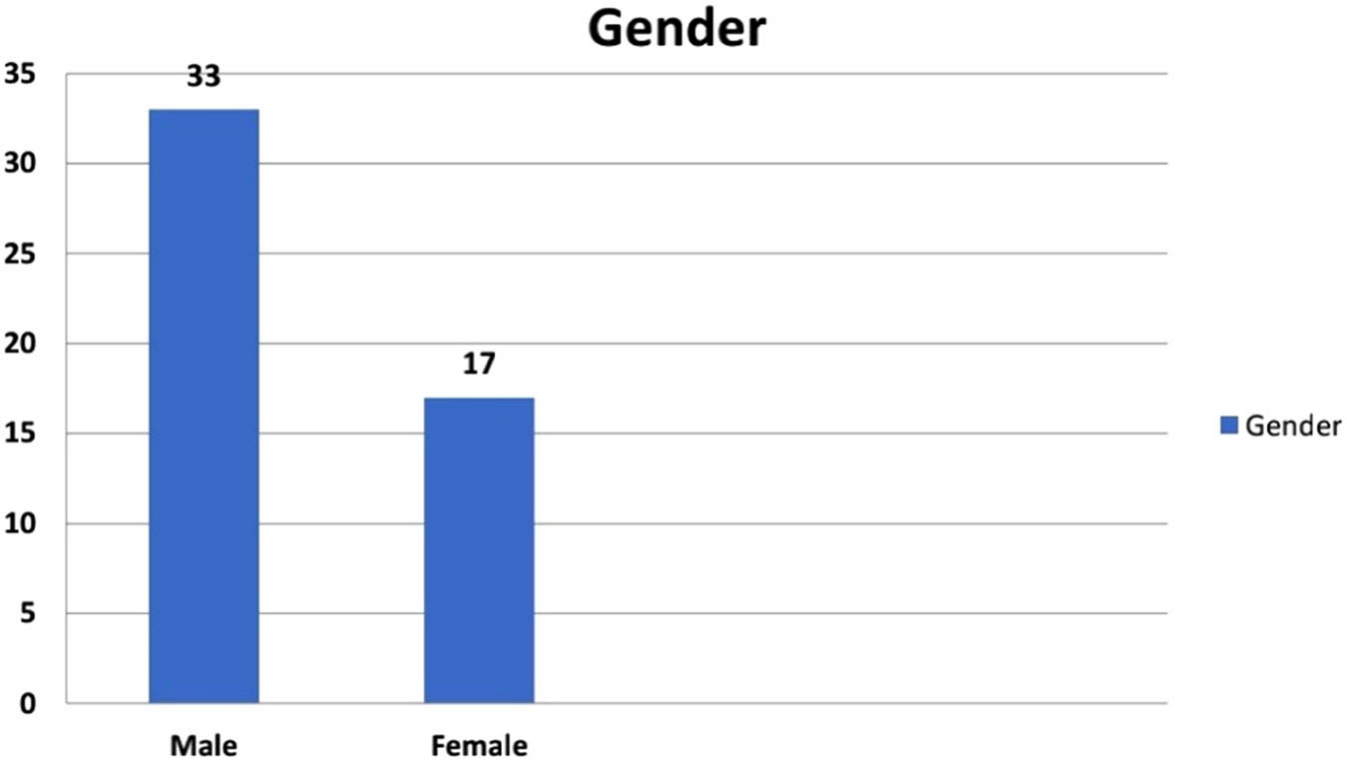
Ratio of male to female patients (*X*‐axis–Gender; *Y*‐axis–Number of participants)

**Figure 4:**
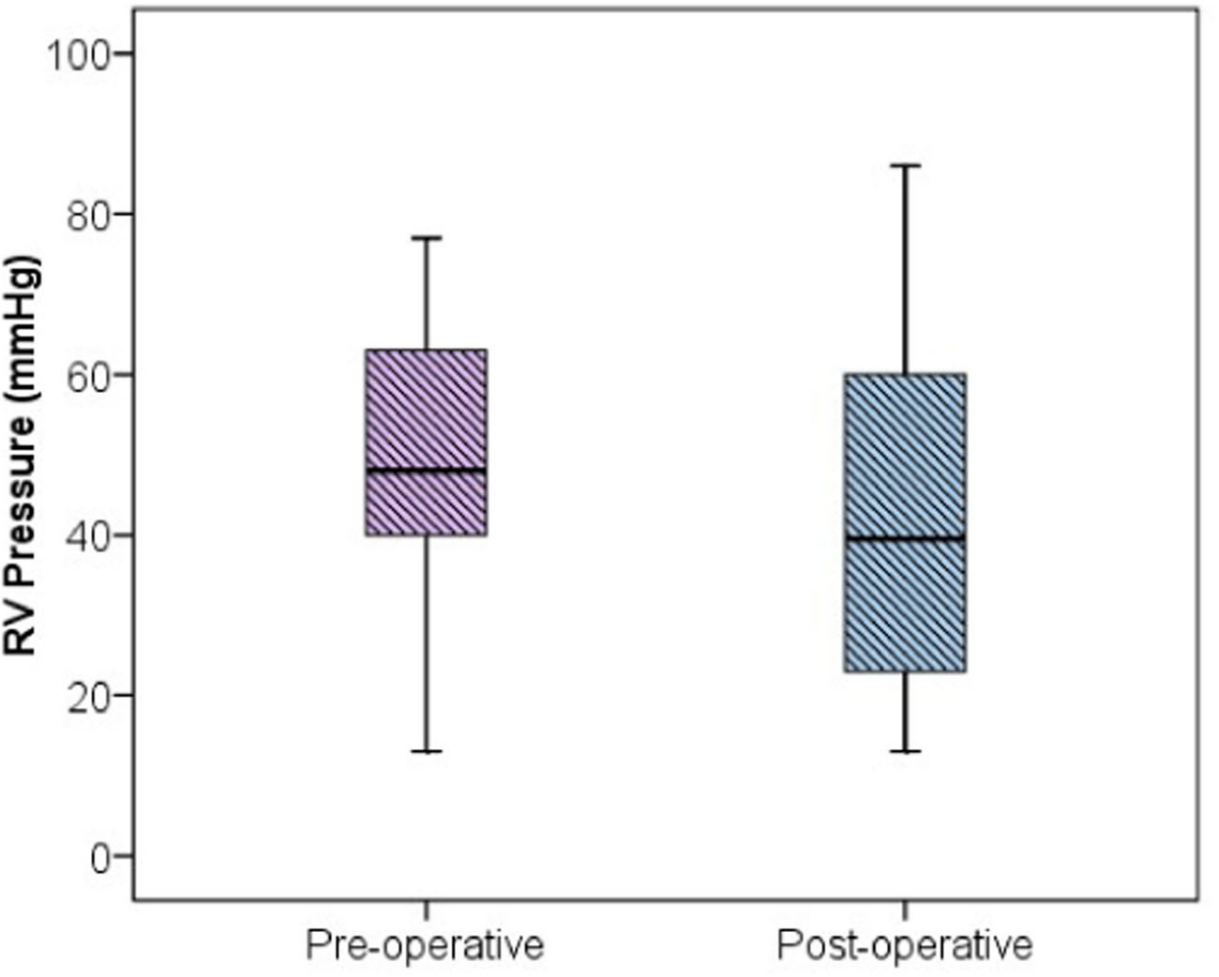
RV pressure (*X*‐axis–Preoperative and postoperative phase; *Y*‐axis–RV pressure in mmHg). RV, right ventricle/right ventricular.

**Figure 5:**
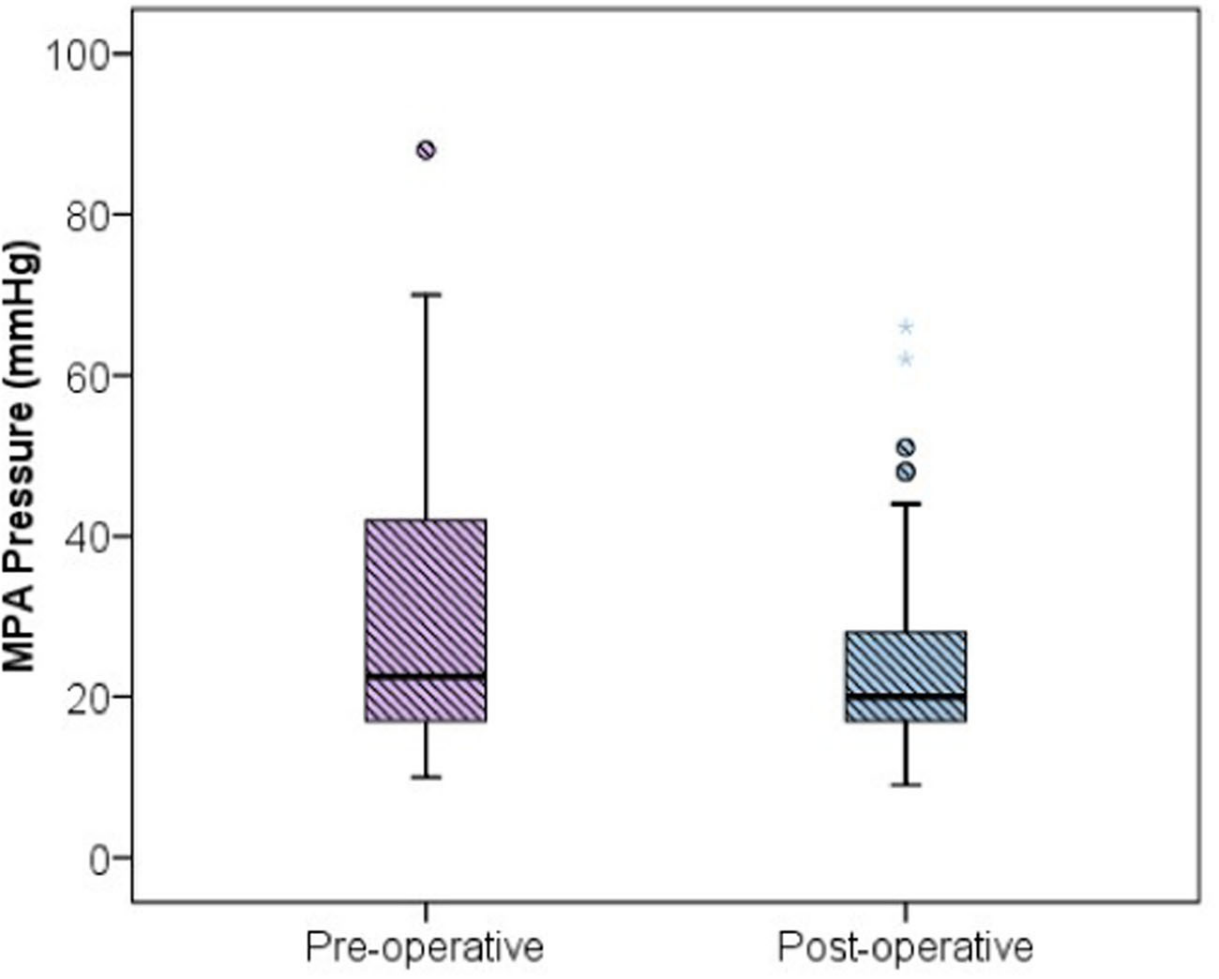
MPA pressure (*X*‐axis–Preoperative and postoperative phase; *Y*‐axis–MPA pressure in mmHg).

**Figure 6:**
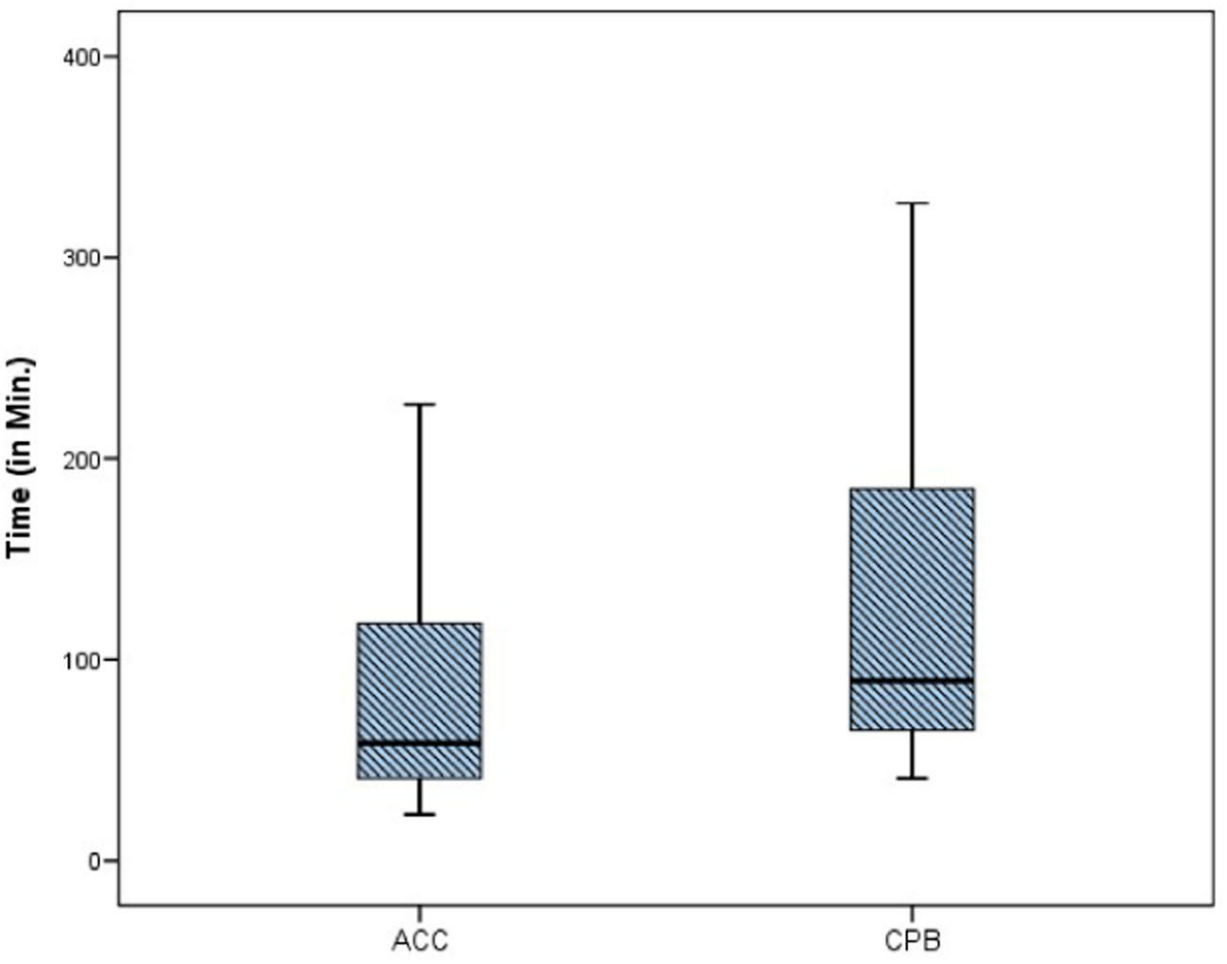
ACC and CPB time correlation. ACC, aortic cross‐clamp; CPB, cardiopulmonary bypass.

**Figure 7:**
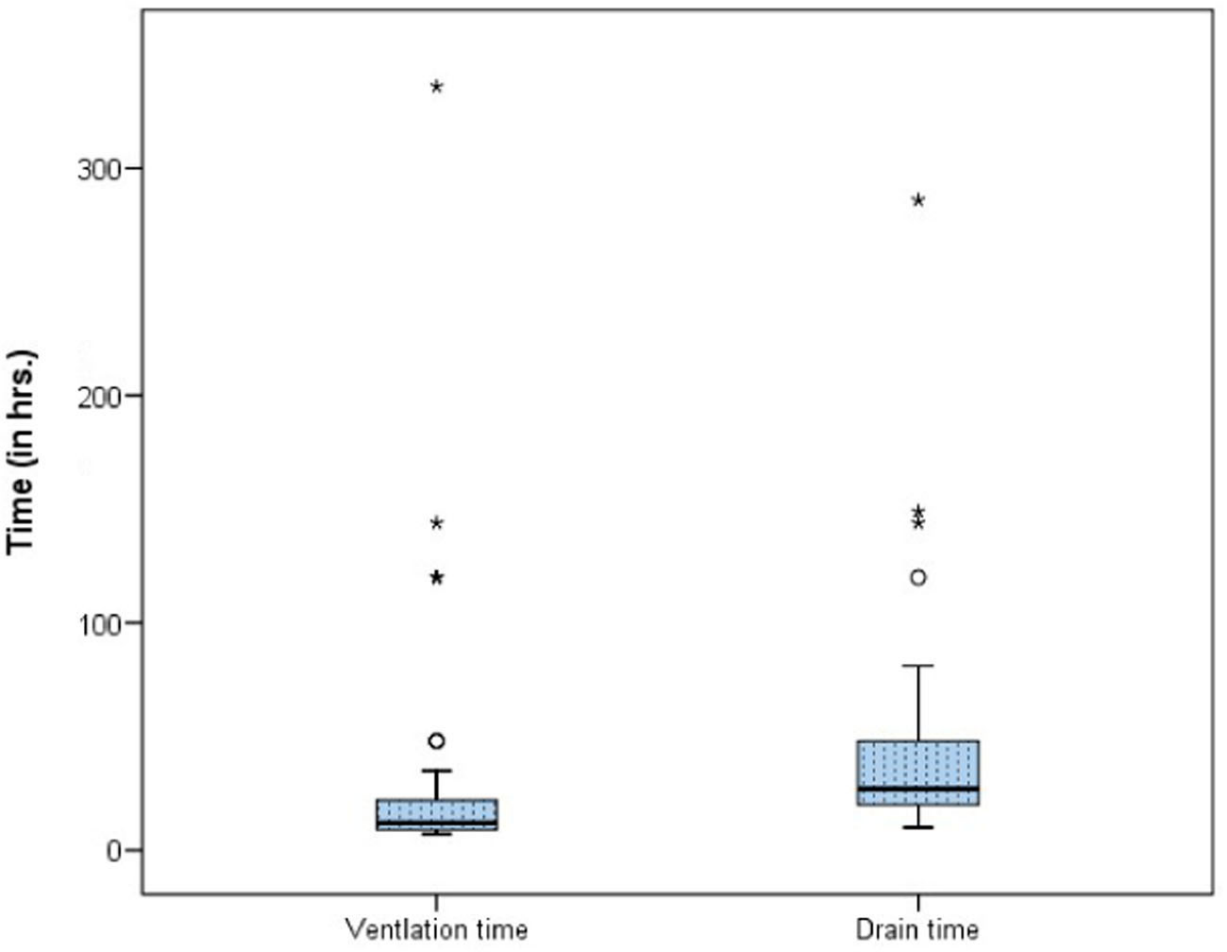
Correlation of ventilation time and mediastinal drainage in hours

**Figure 8:**
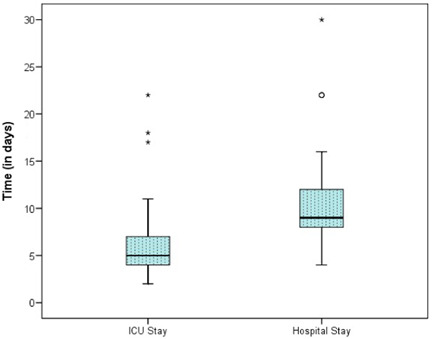
Correlation of ICU stay and hospital stay in days. ICU, intensive care unit.

**Figure 9:**
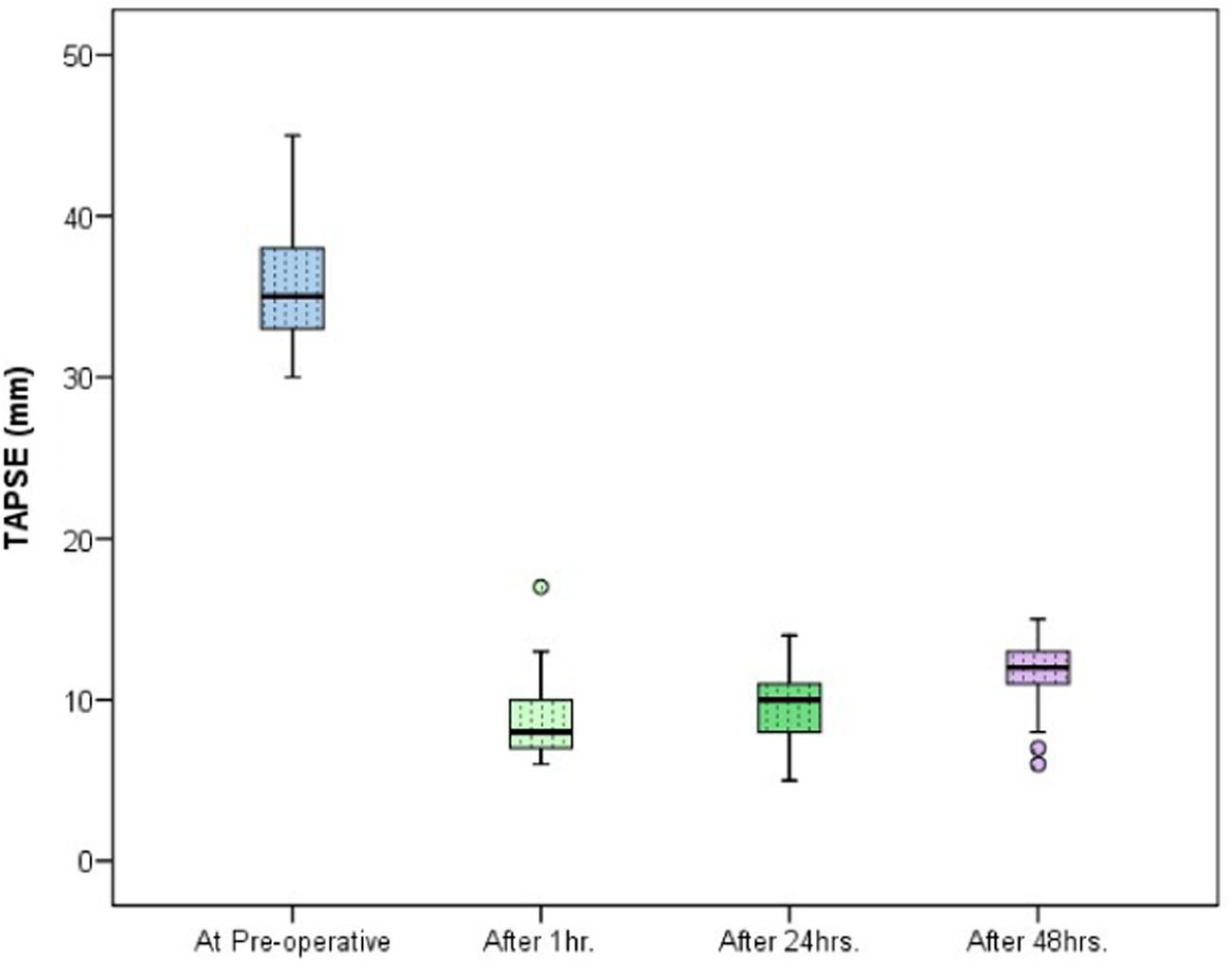
TAPSE value at preoperative, after 1, 24, and 48 h. TAPSE, tricuspid annular planar systolic excursion.

**Figure 10:**
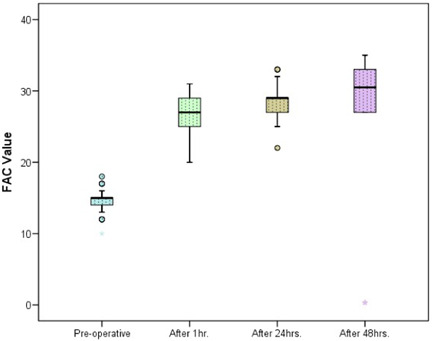
FAC value at preoperative, after 1, 24, and 48 h. FAC, fractional area change.

The demographic data and male:female ratios are represented in Table 4 and Figure 3, respectively. The relevant results concerning the comparison of RV and main pulmonary artery (MPA) pressure preoperatively and postoperatively are outlined in Table 5 and Figures 4 and 5. The correlations between ACC and CPB time, ventilation time, mediastinal drainage, ICU, and hospital stays were also estimated (Figures 6, 7, 8). Similarly, the mean values for TAPSE and FAC were obtained in presurgical and postsurgical phases (Table 6, Figures 9 and 10).

**Table 4:** Demographic data of 50 patients with congenital heart diseases (CHD)

Parameter	Mean ± SD
Age (months)	33.46 ± 40.11
Height (cm)	81.23 ± 23.71
Body surface area (m^2^)	0.43 ± 0.18
Hemoglobin (g%)	11.96 ± 2.02

**Table 5:** Comparison of preoperative and postoperative RV and MPA pressure

Sr. No.	Variable	Preoperative (*n* = 50); mean (SD)	Postoperative (*n* = 50); mean (SD)	*p* Value
1	RV pressure mmHg	49.1 (16.1)	42.7 (2.9)	<0.002
2	MPA pressure mmHg	30.4 (2.6)	24.2 (12.9)	<0.001

Abbreviation: RV, right ventricle/right ventricular.

**Table 6:** Comparison of TAPSE and FAC preoperative and postoperative at different time

Sr. No.	Variable	Preoperative (*n* = 50)	Postoperative after 1 h (*n* = 50)	*p* Value
1	TAPSE mean (SD), mm	35.6 (3.5)	8.8 (2.2)	<0.001
2	FAC mean (SD), mm	14.4 (1.5)	26.5 (2.4)	<0.001

Sr. No.	Variable	Preoperative (*n* = 50)	Postoperative after 24 h (*n* = 50)	
1	TAPSE mean (SD)	35.6 (3.5)	9.8 (1.9)	<0.001
2	FAC mean (SD)	14.4 (1.5)	28.5 (2.2)	<0.001

Sr. No.	Variable	Preoperative (*n* = 50)	Postoperative after 48 h (*n* = 50)	
1	TAPSE mean (SD)	35.6 (3.5)	11.7 (2.1)	
2	FAC mean (SD)	14.4 (1.5)	25.1 (12.6)	<0.001

Abbreviations: FAC, fractional area change; TAPSE, tricuspid annular planar systolic excursion.

According, to the postoperative echocardiographic data, the mean ± SD of TAPSE and FAC was 35.62 ± 3.59 and 14.4 ± 1.5 at baseline as compared with 8.80 ± 2.2 and 26.5 ± 2.40 at 1 h postoperatively respectively. However, the difference was statistically significant. The mean ± SD of TAPSE and FAC was 8.82 ± 2.21 and 26.5 ± 2.40 at 1 h postoperatively and 9.8 ± 1.9 and 28.5 ± 2.19 at 24 h postoperatively, respectively; the difference was statistically significant. The mean ± SD of TAPSE and FAC was 9.8 ± 1.9 and 28.5 ± 2.19 at 24 h postoperatively as compared with 11.7 ± 2.02 and 25.1 ± 12.6 at 48 h postoperatively, respectively. This comparison was statistically significant. The mean ± SD of TAPSE and FAC was 35.62 ± 3.59 and 14.4 ± 1.5 at baseline as compared with 25.1 ± 12.6 and 11.7 ± 2.1 at 48 h postoperatively, respectively, which was a statistically significant difference. From the echocardiographic data, the difference in CPB time between 1, 24, and 48 h postoperatively was statistically significant. The difference in ACC time at these same time was also significant.

At 1 h after the operation, all 50 patients had RV dysfunction, and after 48 h, RV function was poor and additionally increased. In addition, TAPSE was a more accurate echocardiographic parameter than FAC.

## 6 DISCUSSION

The primary goal of this study was to use recognized deformational RV techniques to characterize and predict the acute intraoperative change in RV function. Improvements in surgical procedures and advances in sedation and critical care have increased postoperative cardiac endurance. Many factors have been attributed to the poor postoperative cardiac outcome, including underlying defect with a small primary PA annulus, extreme hypoplasia or insufficient right or left PA, ventriculotomy and RV outflow patch, myocardial hypoxia during CPB, or ARDS.^20^ Despite a favorable intracardiac repair (ICR), some patients have a challenging postoperative course marked by delayed breathing and inotropic assistance. RV dysfunction has been observed in these patients in the past (low heart yield, high focal venous filling pressure, expanded inotropic prerequisite, and delayed ventilation). The discovery of hazard elements to distinguish this subset of patients considers a better allocation of medical clinic assets, further developed results, and significantly reduced medical clinic expenditures.

### 6.1 Incidence of RV dysfunction

In the early postoperative period, RV dysfunction is described by elements of prohibitive physiology on Doppler echocardiography (presence of an antegrade diastolic pulmonary flow coinciding with atrial systole).^21^, ^22^ According to the literature, its popularity ranges from 28% to 63%.^23^, ^24^ In our study, during the postoperative transitional period (1, 24, and 48 h), most patients had RV dysfunction (restrictive physiology) instantly following cardiac surgery. The high occurrence in our study can be justified as follows: (1) a transannular patch was used in all patients in this study, as a correlation has been established between this technique and the appearance of anterograde diastolic stream in the PA, and (2) tissue Doppler (along with 2D pulse Doppler) was employed to evaluate RV function, which is more delicate than 2D pulse Doppler.^23^, ^25^, ^26^

Multiple processes such as increased RV afterload due to pulmonary hypertension affect RV contractility in cases of idiopathic dilated cardiomyopathy (DCM). RV is also affected by cardiomyopathic activity such as ventricular interdependence caused by septal dysfunction and myocardial ischemia due to lessened coronary perfusion. However, RV systolic dysfunction is believed to be a conventional final pathway in heart failure, which may lead to a poor prognosis.^27^, ^28^, ^29^

Tricuspid regurgitation forth and indications of foundational venous blockage, like raised jugular venous tensions, foresee higher mortality in patients with cardiovascular breakdown.^30^ Excessive right atrial pressure is linked to hepatic and renal dysfunction, leading to malnutrition and cardiorenal syndrome.^31^ Because of the related bleakness and mortality as well as abrupt cardiovascular passing in patients with left‐sided cardiovascular breakdown, risk stratification in DCM is significant.^32^ Due to the related bleakness and mortality as well as abrupt cardiovascular passing in patients with a left‐sided cardiovascular breakdown of the significance of RV systolic dysfunction on both morbidity and mortality.^33^

CMRI is recommended as the gold standard approach for RV evaluation in guidelines; however, it is not readily available, which is seen as a serious limitation to its use.^34^ Nonetheless, echocardiography is a noninvasive, inexpensive, and widely available way of assessing RV performance. Consequently, transthoracic echocardiography is the most common imaging tool for RV evaluation. Despite this, precise RV shape and capacity measurements are challenging because of the RV's complicated structure. Thus, in this study, we reviewed the echocardiographic modifications of RV aspects and capacity related to persistent cardiovascular breakdown and to foresee the predominance of RV systolic dysfunction in patients with constant cardiovascular breakdown, given echocardiographic boundaries.

Singh et al.^35^ assessed RV function in postoperative patients with Tetralogy of Fallot and its predictive factors. Patients who were allocated into group A (with RV dysfunction) and group B (without RV dysfunction), underwent preoperative and postoperative clinical evaluation, biochemical assessment, and 2D echocardiography (pulse and tissue Doppler). Echocardiography was performed at different intervals after ICR. Patients were also evaluated for intraoperative parameters such as cross‐clamp and bypass time besides acidosis, in addition to RV and PA pressure. All of them underwent ICR (transannular patch repair). Only 54.2% (*n* = 13/24) of patients demonstrated RV dysfunction. After 12 weeks, only 15.3% (*n* = 2/13) still present the dysfunction. The presence of cyanotic spell (*p* = 0.006), recurrent chest infection (*p* = 0.002), increased hematocrit (*p* = 0.02), and increase in serum iron level (*p* = 0.002) were considerably correlated with postoperative RV dysfunction, resulting in extended ICU stay and slower recovery. Preoperative RV dysfunction was related to difficult weaning from bypass and poor postoperative outcomes (*p* < 0.001). Singh et al. concluded that RV dysfunction is frequently present even after adequate ICR. However, in this setting, its etiology remains to be established. In this study, the preoperative clinical profile and fluctuations in serum iron levels were important contributing factors to postoperative outcomes.

Chaturvedi et al.^36^ demonstrated that restrictive dysfunction in the postoperative period was associated with greater intraoperative myocardial injury and postoperative oxidative stress besides in addition to severe iron loading of transferrin. They revealed a positive correlation among iron concentration, troponin T level, and postoperative RV dysfunction. In their study, the iron profile of all their patients suggested a decrease in total iron‐binding capacity (TIBC), boosted serum iron levels, improved transferrin saturation, and increased creatine kinase myocardial band (CK‐MB) levels in RV dysfunction patients. The acute‐phase response due to CPB includes decreased TIBC and transferrin, which has been attributed to the extravasations of these proteins. A pattern of iron overload superimposed on this acute‐phase response results in an increase in the availability of free iron, which acts as a catalytic agent for free radical production. A low TIBC along with high serum iron raises the level of free iron. However, in this study, the mean levels of serum iron only reached the high normal value. Ferritin, on the other hand, is an acute‐phase reactant protein that increases as part of the inflammatory response. The continuous inflammatory response generated by CPB stress could explain the large increase in ferritin in both groups, which did not significantly differ between them in the postoperative period (*p* = 0.41). Though, only RV dysfunction patients had an iron overload. The myocardium of the tetralogy of Fallot lacks antioxidants, making it susceptible to free radical‐mediated injury and leading to impaired RV functions. The CK‐MB levels were significantly higher in patients who acquired postoperative RV dysfunction. That indicates that myocardial impairment in patients after ICR is significantly greater in patients with RV dysfunction than in those patients with a normal postoperative course.

In heart failure, Kjaegaard et al.^37^ investigated the determinants of RV function as evaluated using TAPSE. TAPSE has unbiased predictive value in individuals with heart failure; however, LV ejection fraction may influence it. By analyzing 634 patients with symptomatic heart failure, this study looked at the relationship between TAPSE and the clinical variables of global and regional LV function. TAPSE was linked to global and regional longitudinal LV function assessments, segmental wall motion scores, and diastolic LV function as evaluated by transthoracic echocardiography. TAPSE was significantly linked to LV ejection fraction, wall motion index score, and atrioventricular annular plane systolic excursion of the mitral annulus. However, septal and posterior mitral annular plane systolic excursion (*B* = 0.56, *p* < 0.0001, and *B* = 0.35, *p* = 0.0002 per mm, respectively) and nonischemic etiology of heart failure (*B* = 1.3, *p* = 0.002) were independent predictors of TAPSE (*R* ^2^ = 0.28, *p* < 0.0001). TAPSE's predictive value was not related to the cause of heart failure or any of the other clinical parameters studied. The authors thought that TAPSE might have diminished in heart failure individuals with heart failure with LV dysfunction, especially those who have reduced longitudinal septal motion. TAPSE levels were also lower in patients with ischemic heart failure. However, the absolute decrease in TAPSE was minimal. It appears to have no major impact on TAPSE's clinical use, whether as a marker of RV systolic function or as a predictive factor.

### 6.2 Preoperative factors and their relationship with RV dysfunction and the demographic profile of patients developing RV dysfunction

#### 6.2.1 Age

Repair at a young age has been shown to minimize postoperative RV dysfunction caused by persistent hypoxia and stress loading, which causes the substrate in the myocardium that is liable for arrhythmias and diastolic brokenness in patients going through late repair. Significant RV remodeling has been predicted if repairs are carried out before the age of 6 months. It has been suggested that irreversible changes are set in by 4 years of age, based on microscopic analysis; consequently, repair before the age of 3 years is optimal. According to the research above, repairs performed before 3 years of age may cause myocardial changes to recur. Still, the best functional outcome is attained before 1 year of age when changes are just starting to emerge. Therefore, at 3 years of age, the foundation of irreversible changes, “age” may have decreased the effect on postoperative outcomes.

### 6.3 Preoperative hemoglobin

According to the literature, more than 18 g% hemoglobin level has been linked to serious disease. RV physiology can be unreliable preoperatively or postoperatively. It is unclear whether this is part of the same pattern or if the same functional substrate is involved. Diastolic dysfunction may indicate greater ischemia myocardium, hence a higher risk of reperfusion injury or ischemic offense.

In this prospective observational study, we examined the demographic attributes and other factors of 50 patients. The difference between preoperative and postoperative RV and PA pressure was statistically significant with *p* values of <0.002 and <0.001, respectively.

The management of the CPB machine and the CP emulsion directly influence postoperative states. Davidson et al.^38^ noticed that expanded doses of CP were correlated with greater degrees of enhanced left anterior descending coronary artery flow after congenital cardiac surgery. According to a meta‐analysis of 5879 patients, warm CP was associated with a higher postoperative cardiac index than cold one. However, warm CP alludes to other possible influences such as intermittent versus continuous, antegrade versus retrograde, and composition of CP.^31^ CPB perfusion temperature is an additional variable that impacts postoperative patient conditions. Tönz et al.^39^ realized that systemic vascular resistance at 3 and 9 h after CPB was higher in patients who underwent hypothermic CPB than in those who underwent the normothermic one. In our study, we observed a significant difference in the intraoperative variables of total CPB time and ACC time. Whenever CPB and ACC times increased during the surgical procedure as compared with postoperatively, the patients' ventilation hours, mediastinal chest drainage, ICU stay, and hospital stay also increased.

## 7 CONCLUSION

After ICR, RV dysfunction causes a long recovery time and a delayed postoperative course. Preoperative RV diastolic dysfunction could be another marker for distinguishing populations with a higher risk of postoperative mortality or requiring vasoactive support and those with difficulty weaning from bypass.

CPB time is directly proportional to RV dysfunction that commonly occurs after ICR and is transient and resolves in the maximum number of patients after 48 h. The application of TAPSE and FAC pre‐ and postoperatively enables the evaluation of to evaluate postoperative outcomes (ICU stay and, mechanical ventilation time) effectively, whereas TAPSE proved to provide a more accurate estimate of RV function when compared than FAC.

## 8 LIMITATIONS

This study has limitations, such as the small sample size from a single center and the lack of follow‐up data to evaluate the effect of RV systolic dysfunction on survival. The patients enrolled in the study were assessed by a pediatric cardiologist and designated echocardiographer who were coauthors in the study. Further studies involving a greater sample size, and long‐term follow‐up data will be required to better understand the effect of RV systolic dysfunction on survival rate.

## AUTHOR CONTRIBUTIONS

**Vishal V. Bhende**: Conceptualization; data curation; formal analysis; funding acquisition; investigation; methodology; project administration; resources; software; supervision; validation; visualization; writing–original draft; writing–review and editing. **Tanishq S. Sharma**: Conceptualization; data curation; formal analysis; funding acquisition; investigation; methodology; project administration; resources; software; supervision; validation; visualization; writing–original draft; writing–review and editing. **Bhadra Y. Trivedi**: Data curation; formal analysis; investigation; methodology; project administration; resources; software; supervision; validation; visualization. **Amit Kumar**: Data curation; formal analysis; investigation; methodology; project administration; software; supervision; validation; visualization. **Dushyant M. Parmar**: Data curation; formal analysis; funding acquisition; investigation; methodology; project administration; resources; software; supervision; validation; visualization. **Paresh Nerurkar**: Data curation; formal analysis; investigation; methodology; project administration; resources; software; supervision; validation; visualization. **Prachi M. Shah**: Data curation; formal analysis; investigation; methodology; project administration; resources; software; supervision; validation; visualization. **Naresh J. Fumakiya**: Data curation; formal analysis; investigation; methodology; project administration; resources; software; supervision; validation; visualization. **Hardil P. Majmudar**: Data curation; formal analysis; investigation; methodology; project administration; resources; software; supervision; validation; visualization. **Sohilkhan R. Pathan**: Data curation; formal analysis; investigation; methodology; project administration; resources; software; supervision; validation; visualization.

## CONFLICT OF INTEREST

The authors declare no conflict of interest.

## TRANSPARENCY STATEMENT

The lead author Vishal V. Bhende affirms that this manuscript is an honest, accurate, and transparent account of the study being reported; that no important aspects of the study have been omitted; and that any discrepancies from the study as planned (and, if relevant, registered) have been explained.

## Data Availability

The data that support the findings of this study are openly available in [Authorea] at https://doi.org/10.22541/au.164304198.80688551/v1] [reference number].
